# *In vitro* metabolism studies of erythraline, the major spiroalkaloid from *Erythrina verna*

**DOI:** 10.1186/1472-6882-14-61

**Published:** 2014-02-18

**Authors:** Thais Guaratini, Denise Brentan Silva, Aline Cavalli Bizaro, Lucas Rossi Sartori, Hans-Ulrich Humpf, Norberto Peporine Lopes, Letícia Veras Costa-Lotufo, João Luis Callegari Lopes

**Affiliations:** 1 Núcleo de Pesquisa em Produtos Naturais e Sintéticos (NPPNS), Faculdade de Ciências Farmacêuticas de Ribeirão Preto (FCFRP), Universidade de São Paulo (USP), Av. Café s/nº, 14040-903 Ribeirão Preto, SP, Brazil; 2 Lychnoflora Pesquisa e Desenvolvimento em Produtos Naturais LTDA, Rua Ângelo Mestriner 263, Ribeirão Preto, SP, Brazil, Ribeirão Preto, SP, Brazil; 3 Institute of Food Chemistry, Westfälische Wilhelms-Universität Münster, Corrensstrasse 45, 48149 Münster, Germany; 4 Departamento de Fisiologia e Farmacologia, Faculdade de Medicina, Universidade Federal do Ceará, 60430-270 Fortaleza, CE, Brazil

**Keywords:** Spirocyclic alkaloids, Erythraline, *in vitro* metabolism, *Erythrina verna*, Fabaceae, Jacobsen catalyst, Erythrina alkaloids

## Abstract

**Background:**

*Erythrina verna*, popularly known as “mulungu”, is a Brazilian medicinal plant used to treat anxiety. Erythrina alkaloids have been described in several species of *Erythrina*, which have biological and therapeutic properties well known that include anxiolytic and sedative effects.

**Methods:**

In this work, *in vitro* metabolism of erythraline (1), the major spirocyclic alkaloid of *Erythrina verna*, was studied in the pig cecum model and by biomimetic phase I reactions. The biomimetic reactions were performed with Jacobsen catalyst to produce oxidative metabolites and one metabolite was isolated and evaluated against cancer cells, as HL-60 (promyelocytic leukemia), SF-295 (Glioblastoma) and OVCAR-8 (ovarian carcinoma).

**Results:**

Erythraline exhibited no metabolization by the pig microbiota and a main putative metabolite was formed in a biomimetic model using Jacobsen catalyst. This metabolite was isolated and identified as 8-oxo-erythraline (2). Finally, erythraline and the putative metabolite were tested in MTT model and both compounds showed no important cytotoxic activity against tumor cells.

**Conclusions:**

The alkaloid erythraline was not metabolized by intestinal microbiota, but it was possible to identify its oxidative metabolite from biomimetic reactions. So these data are interesting and stimulate other studies involving this alkaloid, since it is present in phytomedicine products and there are not reported data about the metabolism of erythrina alkaloids.

## Background

**Figure 1:**
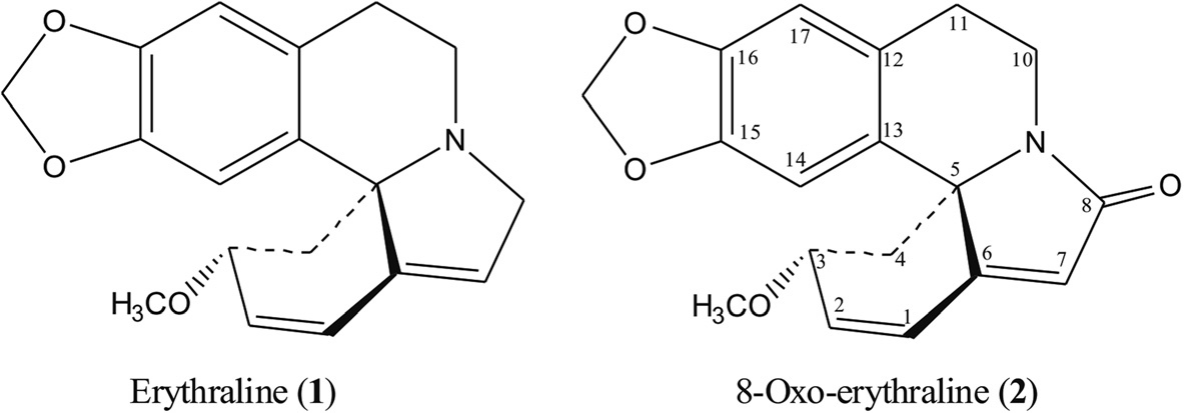
Structures of erythraline (1) and its putative metabolite (2).

The genus *Erythrina* (Fabaceae) comprises 115 species distributed in tropical and subtropical Forest [1]. “Erythros” has Greek origin that means red and is related to the color of the flowers [2]. Erythrina alkaloids are characteristic of this genus with over one hundred structural derivatives described to date [3]–[7]. *E. verna* is one of the 11 species that occur in Brazil and it has being previously classified as *E. mulungu*. In the folk medicine, these plants are used in Brazil as a sedative, to treat sleep disorders and anxiety [8]. Systematic studies have supported the popular use by confirming the anxiolytic effects of a series of erythrina alkaloids [9]–[11] and recently a mechanism of action was proposed as a potent antagonist of α4β2 nicotinic receptors. These observation also could be useful to provide a rational basis for product standardization and for dosing recommendations [12]. Take together, all these previous reports stimulate the development of some phytomedicine products that appear now in the Brazilian market, but to date there are no literature data regarding pharmacokinetics and metabolism of active compounds. Considering these problems, investigations of cultivars that storage erythrina alkaloids in high amounts are fundamental for a pre-clinical trial and recently it was describe a commercial sample that accumulates high levels of erythraline (Figure 1) [13]. In addition, previous investigation described a weak cytotoxicity for Erythrina alkaloids [14], which suggest also the necessity to confirm the cytotoxicity effects of pure compounds and justify the isolation for all assays.

The definition of possible metabolites in gut microbiota, and phase 1 and 2 metabolism reactions are important pre-clinical steps for medicines intended for oral delivery [15]–[17]. The “pig cecum model” was developed and further improved, showing good results for the metabolization of flavonoids [18,19], but no reactions were observed for lignans [20] suggesting, in this case, no metabolization by the gut microbiota. These data are important for further definitions of the compounds pharmacokinetics studies and to stimulate a continuous investigation of possible phase 1 and 2 metabolization products. Several *in vitro* biomimetic models were developed for phase I metabolism investigations. Our group recently applied Jacobsen catalyst ([Mn (salen)]) to produce similar metabolites of CYP450 enzymes yielding the product in good scale [21,22]. Comparative analysis confirmed the presence of the same putative metabolite in biomimetic oxidative model, microsomal fractions and *in vivo* experiments, reinforcing the importance of this methodology for pre-clinical trials [20,23,24]. In this context, the aim of this work was to investigate the metabolism of erythraline in the pig cecum model and through biomimetic reactions, in order to improve information on its pre-clinical pharmacokinetic and also on the biological activity of the putative metabolite.

## Methods

### Plant material and extraction

*E. verna* were acquired commercially of a registered supplier of plant matter. 5.4 kg of powered stem bark was extracted by percolation with ethanol and after solvent elimination afforded the ethanolic extract (488.72 g).

### Isolation of erythraline

The ethanol extract (469.76 g) was solubilized in CH_2_Cl_2_:MeOH (8:2) and extracted with 5% HCl (pH 2); the resulting acid fraction was adjusted to pH 10 with NH_4_OH and exhaustively extracted with CH_2_Cl_2_. This extract was concentrated under reduced pressure to yield the alkaloid extract (4.51 g). This extract (4.34 g) was fractionated by column chromatography on silica gel and eluted with hexane:ethyl acetate gradient system (8:2, 7:3, 6:4, 4:6, 1:9) to yield 155 fractions. The fractions 9–25 (875.0 mg) were purified by HPLC to obtain the alkaloid erythraline (367.5 mg), which was submitted to Mn (salen) oxidation procedure.

### Mn (salen) oxidation procedure

All the reactions were performed in a 10 mL glass vessel equipped with a magnetic stirring bar at room temperature. The reactions were performed in dichloromethane using Mn (salen), erythraline (1) and oxidant (PhIO) in the proportion 1:30:30 (μmol). After 24 hours (reaction time), the samples were analyzed by GC-MS. Control reactions were carried out in the absence of catalyst, under the same conditions as the catalytic runs. No products were detected.

### Isolation of oxidative metabolites

The Mn (salen) oxidation reaction was submitted to semipreparative LC using the following conditions: C18 column (Shim-pack Prep-ODS, 5 μm, 20 mm × 25 cm, Shimadzu), flow rate 9 mL/min and acetonitrile (B) and H_2_O (A) both with TFA 0.02% (v/v) as solvents. The elution profile was 0–25 min – 15-65% B, 25–30 min – 25-80% B, 30–32 min – 80-100% B and 32–35 min – 100% B. The compound 8-oxo-erythraline (2) was isolated from this procedure and characterized by MS and NMR.

*8*-*oxo*-*erythraline* (2): brown amorphous solid;^1^H NMR (CDCl_3_, 400 MHz) δ_H_ 6.79 (dd, J = 2.3, 10.2 Hz, H-1), 6.65 (s, H-14), 6.64 (s, H-17), 6.26 (dbr, J = 10.2 Hz, H-2), 6.01 (sbr, H-7), 5.86 (d, J = 1.0 Hz, O-CH_2_-O), 5.83 (d, J = 1.0 Hz, O-CH_2_-O), 3.81 (m, H-3), 3.69 (m, H-10), 3.58 (ddd, J = 3.7, 6.9, 12.4 Hz, H-10), 3.27 (s, –OCH_3_), 3.07 (ddd, J = 7.5, 9.5, 15.8 Hz, H-11), 2.91 (ddd, J = 3.7, 6.9, 15.8 Hz, H-11), 2.73 (dd, J = 4.9, 11.1 Hz, H-4), 1.62 (t, J = 11.1 Hz, H-4); ^13^C NMR (CDCl_3_, 100 MHz) δ_C_ 171.6 (C-8), 157.7 (C-6), 147.2 (C-15), 146.1 (C-16), 136.8 (C-2), 129.5 (C-13), 127.7 (C-12), 123.8 (C-1), 119.7 (C-7), 109.5 (C-17), 105.0 (C-14), 101.2 (O-CH_2_-O), 74.8 (C-3), 67.3 (C-5), 56.5 (OCH_3_), 41.1 (C-10),38.0 (C-4), 27.3 (C-11). ESI MS (pos. ion mode) *m*/*z* 312.1232 [M + H]^+^ (calcd. for C_18_H_18_NO_4_^+^ 312.12303).

### Evaluation of the metabolism by pig cecum model

#### Animals and cecum collection

Two animals (German Landrace or Angler Sattel x Pietrain, 10–12 months old, 120–150 kg weight) used to carry out the pig cecum model experiments were bred without antibiotics on the diet, also called biodynamic conditions. To ensure that the matrix (feces) were not exposed to the air and kept under anaerobic conditions after the slaughtering of the pig, the ceca were firstly ligated in both orifices and stored in an anaerobic jar with Anaerocult A® (Merck) to be transported to the laboratory. The ceca were obtained from fresh slaughtered pigs, which are bred in a biodynamic farm (Gut Wewel, Senden, Germany) and afterwards delivered to the market (as food). Thus, it was not mandatory to be approved by ethical committees, since the ceca are a waste from the farm production.

#### Preparation of the inoculum suspension

All the steps of the inoculum preparation were done under CO_2_ atmosphere inside a hermetic chamber. All the vessels and solutions used were previously flushed with a mixture of the gases N_2_ and CO_2_ (5:1, v/v). The cecum of both animals were opened and the inside content was split in 20 g (±1.0 g) portions in BD Falcon™ tubes (n = 6). Half of these tubes (n = 3) were sterilized (121°C for 15 min at 1.1 bar using an AMB240 autoclave, Astell, Kent, U.K.) to be used as a negative control (DA). The active inoculum (not autoclaved) was denominated as CA for each animal separated. To each of these tubes (n = 6) were added 20 mL of 0.15 M PBS (pH 6.4) containing a trace element solution 0.0125% v/v (13.2 g CaCl_2_ × 2 H_2_O, 10.0 g MnCl_2_ × 4 H_2_O, 1.0 g CoCl_2_ × 6 H_2_O and 8.0 g FeCl_3_ × 6 H_2_O, all of them dissolved in 100 mL of autoclaved deionized water) and a Na_2_S-solution 11.1% (575.9 mg/100 mL of 0.037 M NaOH solution). The suspensions obtained were afterwards filtered through a net lace in order to avoid large particles at the inoculum suspension. All the procedure were done in duplicate for each single animal.

#### Sample incubation

In a 2 mL Eppendorf® tube, 100 μL of 1.0 mM erythraline solution in MeOH was mixed with 900 μL of the inoculum suspension. The samples were prepared in duplicate for each time point (10, 20, 60, 120, 240 and 480 minutes) and incubated at 37°C. The same procedure was applied to DA samples (negative control) and using 1.0 mM quercetin solution as positive control incubated with active inoculum. On this study two animals (cecum 1 and cecum 2, were divide in two cecum 1.1, 1.2, 2.1 and 2.2, n = 4) were used in order to consider the possible differences between the animals microbiota.

#### Inoculum inactivation, sample preparation and chromatographic analysis

Reached the respective incubation time the samples were immediately stored at -80°C in order to stop the possible reactions. Prior to the sample preparation the inoculum was thawed in a water bath at 37°C and 1.0 mL of hydrochloric acid in methanol (1% v/v) was added. The samples were sonicated for 15 minutes and afterwards centrifuged at 20°C and 12000 × *g* for 10 minutes. The supernatant was collected and used for chromatographic analysis. For this analysis were used an High Performance Liquid Chromatograph (HPLC) equipped with Diode Array Detector (DAD) Jasco XLC™, a reversed phase column Nucleodur C18 ISIS, 5 μm, 150 × 2 mm (Macherey-Nagel) and water (A):methanol (B) (both 0.1% formic acid) at the flow 0.4 mL min^-1^. The elution gradient applied was the following: 0 – 2 min (10% B), 2 – 12 min (10 → 100% B), 12 – 14 min (100% B), 14 – 17 min (100 → 10% B) always keeping an equilibration time between each injection of three minutes. The wavelength used for the quantification was 254 nm and the calibration curve showed r = 0.9957.

### MTT assay

Compounds were evaluated for their cytotoxic effect against three cancer cell lines: HL-60 (promyelocytic leukemia), SF-295 (Glioblastoma) and OVCAR-8 (ovarian carcinoma) using the 3-(4,5-dimethyl-2-thiazolyl)-2,5-diphenyl-2*H*-tetrazolium bromide (MTT) assay [25]. Cells were plated into 96-well plates (1 × 10^5^ cells/mL for adherent cells and 3 × 10^5^ for leukemia cells) and cultured for 24 h prior to addition of tested substances. Compounds were screened by addition of concentrations ranging from 0.016 to 5 μg/mL followed by incubation for at 37°C for 72 h. Control groups received 0.1% of vehicle used to diluted the tested substances (DMSO 0.1%). Three hours before the end of the incubation, 150 μL of a stock solution (0.5 mg/mL) of MTT (Sigma-Aldrich Co., Saint Louis, MO, USA) was added to each well. Absorbance was measured using a DTX 880 Multimode multiplate reader (Beckman Coulter Inc., Fullerton, CA, USA).

## Results and discussion

**Figure 2:**
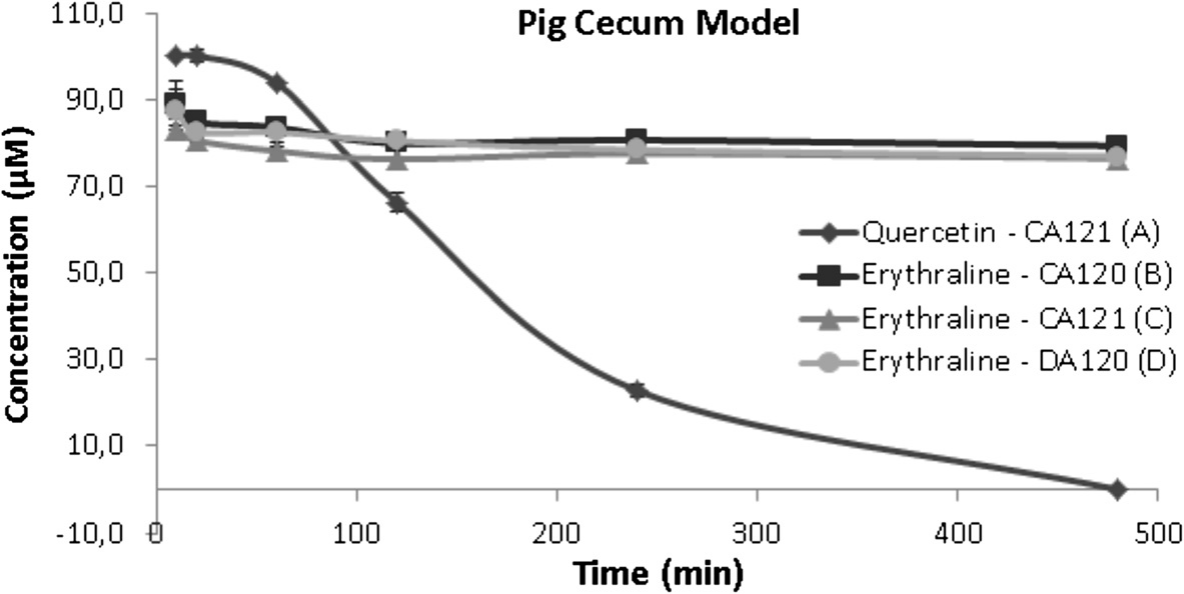
**Degradation curves for erythraline at pig cecum model. (A)** Positive control with quercetin at cecum 2, **(B)** test solution at cecum 1, **(C)** test solution at cecum 2 and **(D)** negative control at cecum 1.

Erythraline (Figure 1) was assayed in pig cecum model and Mn (salen) oxidation procedure as reported before. Under the chosen experimental conditions, no significant degradation of erythraline was observed in the pig cecum model when comparing activated and deactivated ceca after 8 hours of incubation (Figure 2). Even though there are some studies showing metabolism of aromatic natural products in this model [18,19], the yield was very low and apparently the bacteria from the pig intestinal tract are not able to metabolize this alkaloid. The high rate value observed in the metabolization for the positive control using quercetin eliminates any possibility of technical problems. Long-term experiments applying 24 hours of incubation also do not show any significant metabolite formation (Figure 2). This result indicates high intestinal stability of erythraline, which is one prerequisite for good absorption after oral administration.

The high similarity of the bacterial variety between the ceca used can be assumed by the findings already showed on the literature by Hein et al. [19], which used some specific rRNA-based probes in order to quantify the main bacterial groups contained in four ceca. Although the standard deviations of these results are in some cases high, it is possible to note that there is a trend to have similar levels of each bacterial group in all ceca.

Hein et al. [19] also compared their results with the available values for humans, which proves the viability to use the pig cecum model instead the human stool. Another important hint is the origin of the animals used, that were provided by the same biodynamic farm for this study and for the study of Hein et al. [19].

As previously discussed in our recent studies, there is a good correlation between putative *in vitro* and *in vivo* metabolites formation. To investigate the possible erythraline putative metabolites formation in biomimetic models, we applied organometalic catalysis. The proportion between erythraline, Mn (salen) and iodosylbenzene (PhIO) as terminal oxygen donor was 1:30:30 (catalyst/substrate/oxidant) as previously described for lapachol [22]. Excess of PhIO showed a negative influence on the metabolite formation, since most of the erythraline was consumed and decomposed. Using the selected conditions, formation of a major product with acceptable yields after 24 h was observed. The reaction was scaled-up and submitted to isolation process by HPLC allowing us to obtain product (**2**), which was characterized by spectroscopic and spectrometric procedures.

For compound 2, it was observed the ion *m*/*z* 312.1232 [M + H]^+^ as base peak in the mass spectrum obtained by HRESI in positive ionization mode and its molecular formula was determined to be C_18_H_17_NO_4._ The ^1^H NMR spectrum showed signals at δ_H_ 5.83 and 5.86 (*d*, *J* = 1,0 Hz) that characterized the methylenedioxy group (see the spectra in Additional file 1). Further, the other signals indicated a methoxyl group (δ_H_ 3.27) and five olefinic hydrogens, which belongs to the aromatic ring [δ_H_ 6.65 (H-14) and 6.64 (H-17)] and conjugated diene protons [δ_H_ 6.79 (H-1), 6.26 (H-2) and 6.01 (H-7)]. The carbonyl presence at C-8 was confirmed mainly by downfield shift of H-7 (+0.23 ppm) compared with the observed for erythraline and the characteristic chemical shift of the carbonyl (δ_C_ 171.6, conjugated amide). Eleven carbon resonances were observed in DEPT 135° spectra, including six methynes, four methylenes and a methoxyl group. Therefore, the absence of one methylene carbon suggested the oxidation in one of them, as well as the absence of two aliphatic protons (H-8a, H-8b) in the ^1^H NMR when compared with the spectrum of erythraline. Thus, the structure of **2** was determined as 8-oxo-erythraline (Figure 1), which agrees with previously published studies [26].

The final step was to define if erythraline and the putative metabolite showed cytotoxic activity in MTT model. For both compounds 1 and 2, a weak cytotoxicity was observed in the hepatocyte carcinoma Hep-G2 and cervix carcinoma HEP-2 cell lines with IC_50_ values of 17.6 and 15.9 μg/mL for erythraline (1), respectively, and 3.9 and 18.5 μg/mL for 8-oxo-erythraline (2). In the present study, the highest evaluated concentration was 5 μg/mL using a colon adenocarcinoma cell HCT-116, a metastatic melanoma cell MALME-3 M and a metastatic prostate cell PC-3 M, and no significant change on cell viability was observed for both compounds regardless of the cell type used. These data reinforce that those alkaloids could not be considered highly toxic to cancer cell lines, as previously observed for other alkaloids [14,27].

## Conclusions

The results indicated that the alkaloid erythraline is not metabolized by the pig cecum microbiota and one putative metabolite was formed in a biomimetic model using Jacobsen catalyst, which was isolated and identified as 8-oxo-erythraline. Erythraline and its metabolite showed no cytotoxic activity against tumor cells. Our findings are extremely relevant, since erythrina alkaloids are present from phytomedicines used in the therapeutic and there is no information on their metabolism and they could be used for pre-clinical trials.

## Competing interests

The authors declare that they have no competing interest.

## Authors’ contributions

TG, DBS, ACB conducted extraction and isolation of the metabolites, biomimetic reactions and the interpretation of all these data. LRS conducted the study in the pig cecum model and H-UH supervised this study design and interpretation data. LVCL conducted the evaluation of cytotoxicity activity against tumor cell. TG, DBS, NPL and JLCL wrote and revised the manuscript. All authors read and approved the final manuscript.

## Pre-publication history

The pre-publication history for this paper can be accessed here:

http://www.biomedcentral.com/1472-6882/14/61/prepub

## Supplementary Material

Additional file 1 **In vitro metabolism studies of erythraline, the major spiroalkaloid from *Erythrina verna***. Click here for file
