# Supplementary material for: In vitro metabolism studies of erythraline, the major spiroalkaloid from Erythrina verna
Source: BMC Complement Altern Med. 2014 Feb 18;14:61. doi: 10.1186/1472-6882-14-61 (PMC3930555; doi:10.1186/1472-6882-14-61)
Supplement: Additional file 1 — In vitro metabolism studies of erythraline, the major spiroalkaloid from Erythrina verna . [file 1472-6882-14-61-S1.docx]

**Supporting Information**

**In vitro metabolism studies of erythraline, the major spiroalkaloid from *Erythrina verna***

Thais Guaratini^1,2^

Email: thais@lychnoflora.com.br

Denise Brentan Silva^1,2^

Email: denisebrentan@lychnoflora.com.br

Aline Cavalli Bizaro^2^

Email: cavalli.a@hotmail.com

Lucas Rossi Sartori^1^

Email: rossisartori@yahoo.com.br

Hans-Ulrich Humpf^3^

Email: [humpf@uni-muenster.de](mailto:humpf@uni-muenster.de)

Norberto Peporine Lopes^1*^

*Corresponding author

Email: npelopes@fcfrp.usp.br

Letícia Veras Costa-Lotufo^4^

Email: lvcosta@secrel.com.br

João Luis Callegari Lopes^1^

Email: joaoluis@usp.br

^1^Núcleo de Pesquisa em Produtos Naturais e Sintéticos (NPPNS), Faculdade de Ciências Farmacêuticas de Ribeirão Preto (FCFRP), Universidade de São Paulo (USP), Av. Café s/nº, 14040-903, Ribeirão Preto, SP, Brazil.

^2^Lychnoflora Pesquisa e Desenvolvimento em Produtos Naturais LTDA, Campus USP, Ribeirão Preto, SP, Brazil.

^3^Institute of Food Chemistry, Westfälische Wilhelms-Universität Münster, Corrensstrasse 45, 48149 Münster, Germany.

^4^Departamento de Fisiologia e Farmacologia, Faculdade de Medicina, Universidade Federal do Ceará, 60430-270 Fortaleza - CE, Brazil.

**Figure 1S**. ^1^H NMR spectrum of erythraline (**1)** in CDCl_3_ at 300 MHz

**Figure 2S**. ^1^H NMR spectrum of erythraline (**1)** in CDCl_3_ at 300 MHz

**Figure 3S**. ^13^C NMR spectrum of erythraline (**1)** in CDCl_3_ at 75 MHz

**Figure 4S**. DEPT 135° spectrum of erythraline (**1)** in CDCl_3_ at 75 MHz

**
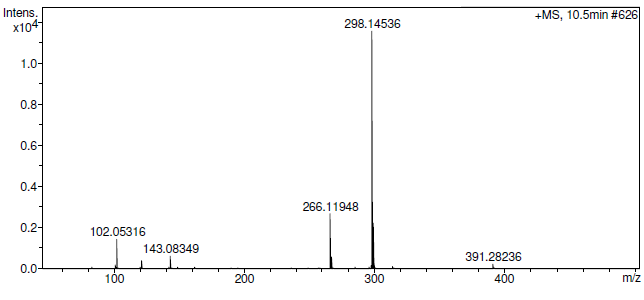
**

**Figure 5S**. HRESIMS spectrum of erythraline (**1**) (positive ionization mode)

**Figure 6S**. ^1^H NMR spectrum of 8-oxo erythraline (**2**) in CDCl_3_ at 300 MHz

**Figure 7S**. ^1^H NMR spectrum of 8-oxo erythraline (**2**) in CDCl_3_ at 300 MHz

**Figure 8S**. ^13^C NMR spectrum of 8-oxo-erythraline (**2)** in CDCl_3_ at 75 MHz

**Figure 9S**. DEPT 135° spectrum of 8-oxo-erythraline (**2)** in CDCl_3_ at 75 MHz


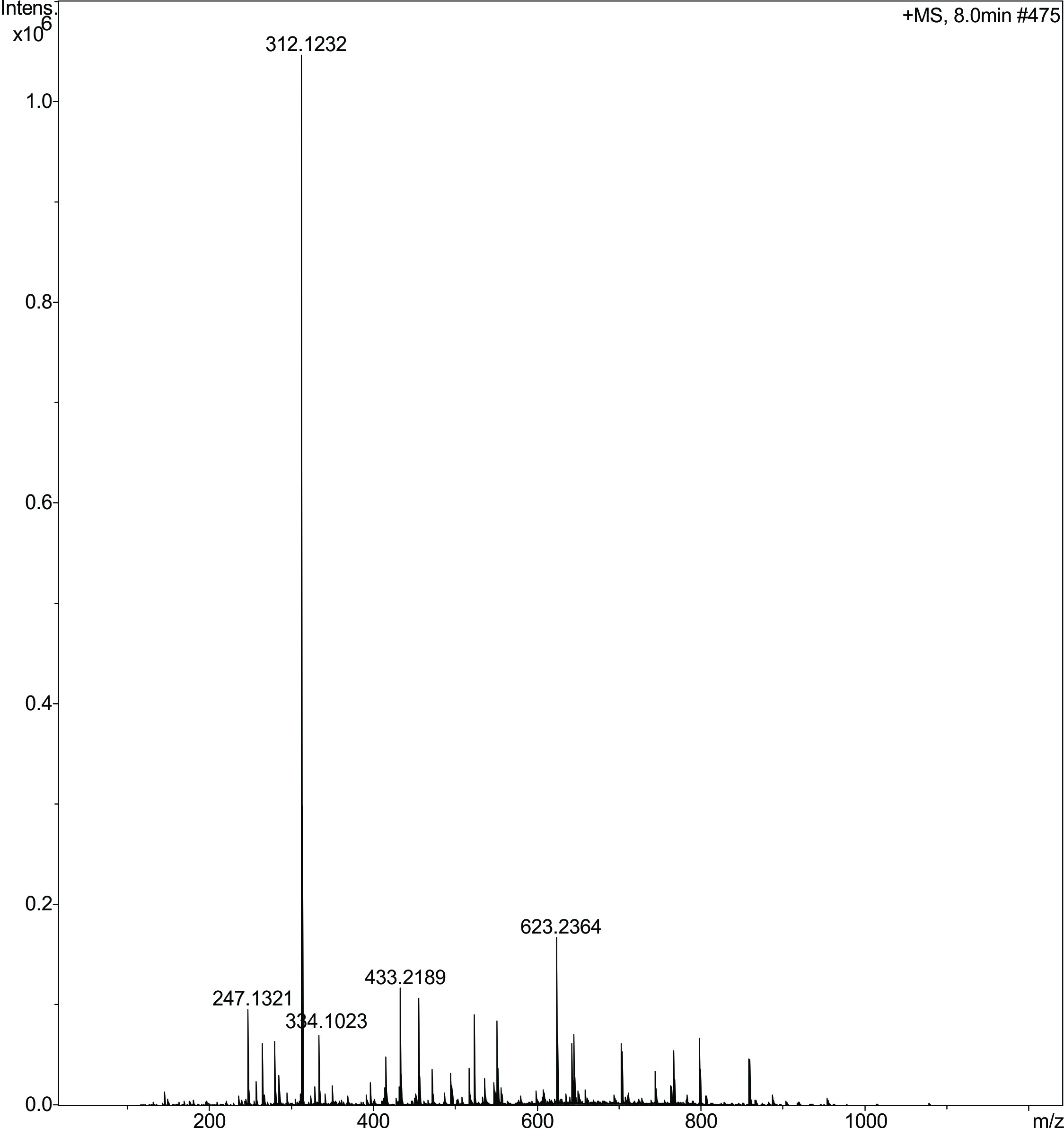


**Figure 10S**. HRESIMS spectrum of 8-oxo-erythraline (**2**) (positive ionization mode)
